# Combined point-of-care biomarkers for risk stratification in patients with non-ST-elevation acute coronary syndrome in the emergency department

**DOI:** 10.3389/fcvm.2025.1711275

**Published:** 2026-02-05

**Authors:** Bo Yan, Zheng Wang, Zhi Liu

**Affiliations:** 1 Emergency Department, Xuanwu Hospital, Capital Medical University, Beijing, China; 2 Emergency Department, Xiongan Xuanwu Hospital, Xiongan New Area, Hebei, China

**Keywords:** acute coronary syndrome, aged, biomarkers, point-of-care testing, risk assessment

## Abstract

**Background:**

Risk stratification is crucial for patients aged ≥60 years with non-ST-elevation acute coronary syndrome (NSTE-ACS). Although the Global Registry of Acute Coronary Events (GRACE) risk score is commonly used, it may be less practical in the emergency setting. This study aimed to evaluate whether a combined point-of-care testing (CM-POCT) model using three biomarkers could effectively predict major adverse cardiovascular events (MACE).

**Methods:**

This retrospective study included 117 patients aged ≥60 years with NSTE-ACS presenting to the emergency department. Point-of-care testing for NT-proBNP, D-dimer, and hs-CRP was performed within 6 h of symptom onset. Independent risk factors were identified, and the predictive value of the three-marker CM-POCT model was compared with the GRACE risk score using receiver operating characteristic curve analysis.

**Results:**

Among the 117 participants (mean age 72.2 ± 8.0 years), 27 experienced MACE. Elevated levels of NT-proBNP (≥2,410 pg/mL), D-dimer (≥0.54 µg/mL), and hs-CRP (≥5.58 µg/mL) were each independent predictors of MACE (all *P* < 0.05). The incidence of MACE increased progressively with the number of elevated markers from 0.0% (no positive markers) to 3.45% (one positive marker), 40.0% (two positive markers), and 86.96% (three positive markers). The area under the curve (AUC) of the three-marker CM-POCT model was 0.958 (95% CI: 0.923–0.993), significantly higher than the GRACE risk score (AUC: 0.822; 95% CI: 0.730–0.913; *P* = 0.003).

**Conclusions:**

The three-marker CM-POCT model could be a more efficient tool for early risk stratification in patients aged ≥60 years with NSTE-ACS in emergency settings. The risk of MACE increases significantly with the number of positive markers.

## 1 Background

Acute coronary syndrome (ACS) affects over 7 million individuals worldwide each year, with non-ST-elevation ACS (NSTE-ACS) accounting for approximately 70% of cases (1). Early risk stratification and timely triage in the emergency department (ED) are essential to prevent major adverse cardiovascular events (MACE) in patients with NSTE-ACS (2). Unlike ST-elevation myocardial infarction (STEMI), where emergency revascularisation is the standard, patients with NSTE-ACS often undergo observation and risk assessment prior to intervention (3). Both the American Heart Association (4) and the European Society of Cardiology (5) guidelines emphasise the importance of revascularisation timing guided by clinical risk stratification. Currently, clinical decisions rely on factors such as the time of symptom onset, electrocardiographic changes, haemodynamics, cardiac biomarkers, and the Global Registry of Acute Coronary Events (GRACE) risk score (6–9).

Although the GRACE risk score is widely endorsed for assessing prognosis in NSTE-ACS (4, 5), its use in emergency settings can be limited by delays in obtaining laboratory results such as cardiac enzymes and serum creatinine. Point-of-care testing (POCT), which refers to bedside testing that delivers rapid results, has emerged as a valuable tool to accelerate clinical decision-making in acute settings (10–12). Previous studies have shown that combining individual biomarkers with GRACE scoring improves MACE prediction (13, 14), and a multimarker strategy—where the presence of multiple elevated markers signals higher risk—has also demonstrated prognostic utility (15, 16). However, research is limited regarding which specific combinations of POCT markers can effectively predict MACE in elderly patients with NSTE-ACS prior to revascularisation.

Elderly patients represent a particularly vulnerable group due to their higher comorbidity burden, atypical symptom presentation, and increased risk of adverse outcomes. Tailored, efficient risk assessment tools are especially important for this population to ensure timely and appropriate management in the ED. To address this gap, we conducted a retrospective study to investigate whether a combined POCT (CM-POCT) strategy using N-terminal pro-B-type natriuretic peptide (NT-proBNP), D-dimer, and high-sensitivity C-reactive protein (hs-CRP) could predict MACE in elderly patients with NSTE-ACS before revascularisation. Additionally, we aimed to compare the predictive performance of CM-POCT with that of the GRACE risk score.

## 2 Methods

### 2.1 Study design and ethical approval

This was a retrospective cohort study conducted in the ED of our hospital between December 2022 and June 2025. The study protocol was approved by the Institutional Ethics Committee (ethical batch number: 2022[161], date: 19/10/2022 and XA[KS2025]003-003, date: 26/11/2025). Owing to the retrospective design, the requirement for written informed consent was waived.

### 2.2 Study subjects

Consecutive patients aged ≥60 years who presented to the ED with symptoms suggestive of ACS were evaluated. Eligible patients were diagnosed with NSTE-ACS—including unstable angina and non-ST-elevation myocardial infarction (NSTEMI)—based on clinical symptoms, electrocardiographic (ECG) changes, and elevated cardiac biomarkers according to current guidelines (4, 5).

The inclusion criteria were (1) age ≥60 years, (2) time from symptom onset to point-of-care testing (POCT) ≤6 h, (3) diagnosis of NSTE-ACS confirmed by guideline-based criteria, and (4) received standardised management during hospitalisation.

The exclusion criteria were (1) pulmonary embolism, aortic dissection, or other thrombotic diseases; (2) cardiomyopathy or moderate-to-severe valvular heart disease; (3) autoimmune, rheumatic, or acute thyroid diseases; (4) use of immunosuppressants or corticosteroids; (5) acute or chronic infection, severe renal dysfunction, or atrial fibrillation that could confound biomarker interpretation; and (6) malignancy, hepatic failure, or refusal of standardised treatment.

### 2.3 Endpoint definition

The primary endpoint was the occurrence of in-hospital MACE prior to revascularisation, which was defined according to international consensus as one or more of the following (4, 5):

- Recurrent or progressive chest pain unresponsive to medication
- Haemodynamic instability or cardiogenic shock
- Life-threatening arrhythmia (including ventricular tachycardia, ventricular fibrillation, pulseless electrical activity, or asystole)
- Acute heart failure
- Dynamic ST-T changes on ECG

The patients were divided into a MACE group or a non-MACE group based on these outcomes.

### 2.4 Laboratory measurements and GRACE risk scoring

Upon admission to the ED, 6 mL of venous blood was collected at the bedside. The first test results were used for analysis. The POCT panel included myoglobin (Myo), creatine kinase-MB (CK-MB), cardiac troponin I (cTnI; conventional, non–high-sensitivity assay; reference range 0–0.04 ng/mL) measured by the AQT90 FLEX immunofluorescence analyser (Radiometer Medical ApS, Brønshøj, Denmark); NT-proBNP (reference range 0–125 pg/mL for <75 years and 0–450 pg/mL for ≥75 years) measured by the Mitsubishi PATHFAST analyser (LSI Medience Corporation, Tokyo, Japan); D-dimer (reference range 0–0.50 µg/mL) and hs-CRP (reference range 0–3 µg/mL); and glycated haemoglobin (HbA1c), all measured using the Wanfu FS-301 immunofluorescence system (Wondfo Biotech Co., Ltd., Guangzhou, China). The POCT turnaround time was <15 min. Additionally, routine blood counts (XN350 analyser) and serum biochemical parameters (Hitachi 7600-210 analyser) were processed within 10 and 90 min, respectively. Sex-specific thresholds were not applied because all participants were aged ≥60 years, and uniform institutional cut-off values were used for analysis.

The GRACE risk score (7, 8) was calculated using the following variables: age, heart rate, systolic blood pressure, serum creatinine (Scr), Killip classification, ECG ST-T changes, cTnI level, and history of cardiac arrest. The patients were classified into three risk groups: low risk (≤108), intermediate risk (109–140), and high risk (>140).

### 2.5 Statistical analysis

All statistical analyses were conducted using SPSS 25.0 (IBM Corp., NY, USA). Continuous variables were expressed as mean ± standard deviation or median with interquartile range; categorical variables were presented as frequencies and percentages. Intergroup comparisons were performed using independent-sample *t*-tests, Mann–Whitney *U* tests, or chi-square tests, as appropriate. Multivariate logistic regression analysis was used to identify independent predictors of MACE. Variables with a *P* < 0.05 in univariate analysis or with established clinical relevance were entered into the multivariate model. A binary logistic regression model was constructed to assess the predictive value of selected laboratory markers. Receiver operating characteristic curve analysis was performed to determine the area under the curve (AUC) and identify the optimal cutoff values of independent predictors using the maximum Youden index. The predictive performance of individual biomarkers, the CM-POCT model, and the GRACE risk score was compared. A two-sided *P* < 0.05 was considered statistically significant.

## 3 Results

### 3.1 Patient selection

A total of 250 consecutive patients aged ≥60 years presenting with suspected NSTE-ACS between December 2022 and June 2025 were initially screened. After applying the predefined inclusion and exclusion criteria, 133 patients were excluded owing to incomplete biomarker data (*n* = 48), recent myocardial infarction (*n* = 32), severe infection or sepsis (*n* = 21), malignancy or severe hepatic/renal dysfunction (*n* = 18), or being lost to follow-up (*n* = 14). The remaining 117 eligible patients were included in the final analysis, of whom 45 (38.46%) were diagnosed with unstable angina pectoris and 72 (61.54%) with NSTEMI. The complete selection process is shown in Supplementary Figure S1.

None of the enrolled patients underwent emergency percutaneous coronary intervention (PCI) at admission; however, 81 (69.23%) subsequently received elective PCI, 29 (24.79%) were managed conservatively with medication after coronary CTA evaluation, and 7 (5.98%) died before PCI due to rapid clinical deterioration. Because the cohort excluded emergency PCI cases, symptom-to-balloon time was not recorded. The in-hospital duration from emergency admission to discharge ranged from 1.5 to 1,113 h, with a median of 162 h.

### 3.2 Comparison of demographics between MACE and non-MACE groups

The mean age of the study cohort was 72.18 ± 8.04 years, and the median time from symptom onset to POCT was 2.5 (1.5–4.5) hours.

Among the 117 participants, 27 (23.08%) developed in-hospital MACE prior to revascularisation. Specific events included recurrent or progressive chest pain unresponsive to medication (*n* = 20), haemodynamic instability or cardiogenic shock (*n* = 10), life-threatening arrhythmia or cardiac arrest (*n* = 7), acute heart failure (*n* = 18), and recurrent dynamic ECG ST-T changes (*n* = 16).

Compared with the non-MACE group, the MACE group had significantly higher values for age, heart rate, hypertension prevalence, Killip classification, GRACE risk score, Myo, CK-MB, cTnI, NT-proBNP, D-dimer, HbA1c, hs-CRP, Scr, leukocytes, and neutrophils (all *P* < 0.05). Conversely, levels of triglycerides, haemoglobin, and serum sodium were significantly lower in the MACE group (Tables 1, 2).

**Table 1:** Participants' demographics between two groups.

Clinical data	All patients (*n* = 117)	MACE group (*n* = 27)	non-MACE group (*n* = 90)	*P*
Female *n* (%)	52 (44.44)	14 (51.85)	38 (42.22)	0.377
Age (year)	72.18 ± 8.04	77.26 ± 7.76	70.66 ± 7.51	**<0** **.** **001**
SBP (mmHg)	143.95 ± 23.82	138.63 ± 30.15	145.54 ± 21.51	0.275
MABP (mmHg)	100.36 ± 14.44	95.79 ± 17.24	101.74 ± 13.29	0.060
HR (bpm)	80 (68, 86)	81 (72, 102)	78 (68, 83)	**0** **.** **021**
CHD *n* (%)	68 (58.12)	19 (70.37)	49 (54.44)	0.141
Hypertension *n* (%)	66 (56.41)	22 (81.48)	44 (48.89)	**0** **.** **003**
Diabetes *n* (%)	21 (17.95)	7 (25.93)	14 (15.56)	0.218
Stroke *n* (%)	6 (5.13)	2 (7.41)	4 (4.44)	0.540
Revascularization *n* (%)	12 (10.26)	1 (3.70)	11 (12.22)	0.201
Hyperhomocystinemia, *n* (%)	30 (25.6)	9 (33.3)	21 (23.3)	0.295
CKD (stage 1–2), *n* (%)	5 (4.3)	2 (7.4)	3 (3.3)	0.397
COPD, *n* (%)	0 (0.0)	0 (0.0)	0 (0.0)	—
Atrial fibrillation, *n* (%)	4 (3.4)	2 (7.4)	2 (2.2)	0.228
Hyperlipidemia, *n* (%)	30 (25.6)	8 (29.6)	22 (24.4)	0.612
Smoking history, *n* (%)	12 (10.3)	4 (14.8)	8 (8.9)	0.392
Obesity, *n* (%)	23 (19.7)	6 (22.2)	17 (18.9)	0.705
Family history of CHD, *n* (%)	6 (5.1)	1 (3.7)	5 (5.6)	0.711
Aspirin use within 1 week, *n* (%)	55 (47.0)	13 (48.1)	42 (46.7)	0.901
Antithrombotic therapy in ED, *n* (%)	61 (52.1)	15 (55.6)	46 (51.1)	0.695
Statin therapy in ED, *n* (%)	30 (25.6)	8 (29.6)	22 (24.4)	0.612
Chronic antihypertensive use, *n* (%)	42 (35.9)	10 (37.0)	32 (35.6)	0.893
Chronic antidiabetic use, *n* (%)	15 (12.8)	4 (14.8)	11 (12.2)	0.748
ECG ST-T *n* (%)	16 (13.68)	6 (22.22)	10 (11.11)	0.141
Killip (grading)	1 (1, 2)	2 (1, 3)	1 (1, 2)	**<0** **.** **001**
GRACE (score)	147.15 ± 43.16	185.07 ± 37.02	135.77 ± 38.21	**<0** **.** **001**
POCT time (h)	2.5 (1.5, 4.5)	3.0 (1.3, 4.5)	2.4 (1.5, 4.5)	0.948

SBP, systolic blood pressure; MABP, mean arterial blood pressure; HR, heart rate; CHD, history of coronary heart disease; ECG ST-T, the ST-T segment changes occurred on the ECG; GRACE, global registry of acute coronary events, POCT time, the time from symptom onset to point-of-care testing.

Bold values indicates statistical significance (*p* < 0.05).

**Table 2:** Laboratory tests comparison between MACE and non-MACE groups.

Biomarkers	MACE group (*n* = 27)	non-MACE group (*n* = 90)	*P*
Myo (ug/mL)	103.0 (69.0, 323.0)	45.5 (19.8, 76.3)	**<0** **.** **001**
CK-MB (IU/mL)	6.70 (4.10, 42.00)	2.32 (2.00, 5.03)	**<0** **.** **001**
cTnI (ng/mL)	0.34 (0.06, 2.60)	0.01 (0.01, 0.17)	**<0** **.** **001**
NT-proBNP (pg/mL)	6,728.0 (1,252.0, 18,418.0)	240.5 (55.5, 757.0)	**<0** **.** **001**
D-dimer (ug/mL)	1.31 (0.77, 2.39)	0.33 (0.21, 0.53)	**<0** **.** **001**
HbAlc (%)	6.0 (5.8, 7.2)	5.7 (5.18, 6.7)	**0** **.** **038**
hs-CRP (mg/L)	15.51 (11.11, 27.20)	2.32 (0.50, 9.64)	**<0** **.** **001**
Hcy (mmol/L)	15.2 (11.0, 21.0)	14.0 (11.0, 18.9)	0.509
TG (mmol/L)	1.16 (0.89, 1.74)	1.70 (1.16, 2.40)	**0** **.** **011**
Scr (μmol/L)	78.0 (58.0, 126.0)	65.0 (53.8, 77.3)	**0** **.** **046**
Glu (mmol/L)	9.55 ± 6.17	7.48 ± 2.82	0.102
Leu (×10^9^/L)	9.56 ± 3.51	7.63 ± 2.51	**0** **.** **012**
Neu (×10^9^/L)	6.77 ± 3.04	5.22 ± 2.31	**0** **.** **019**
Hb (×10^12^/L)	123.63 ± 20.70	135.02 ± 18.14	**0** **.** **007**
Plt (×10^9^/L)	227.07 ± 87.28	206.23 ± 61.66	0.167
TCh (mmol/L)	4.37 ± 1.05	4.35 ± 1.18	0.911
LDL-C (mmol/L)	2.77 ± 1.03	2.68 ± 1.00	0.694
Apo-B (g/L)	0.82 ± 0.21	0.80 ± 0.23	0.806
UA (μmol/L)	364.96 ± 144.40	338.82 ± 101.81	0.386
K^+^ (mmol/L)	4.19 ± 0.51	4.13 ± 0.40	0.507
Na^+^ (mmol/L)	137.43 ± 6.81	140.22 ± 2.94	**0** **.** **047**

Myo, myohemoglobin; CK-MB, creatinine kinase MB; cTnI, cardiac Troponin I; NT-proBNP, N-terminal pro-B type natriuretic peptide; HbAlc, glycosylated hemoglobin; hs-CRP, hypersensitive C reactive protein; Hcy, homocysteine; TG, triglyceride; Scr, serum creatinine; Glu, glucose; Leu, leukocyte; Neu, neutrophile granulocyte; Hb, hemoglobin; Plt, platelet; TCh, total cholesterol; LDL-C, low density lipoprotein-cholesterol; Apo-B, apolipoprotein-B; UA, uric acid; K^+^, kalium ion; Na^+^, natrium ion.

Bold values indicates statistical significance (*p* < 0.05).

### 3.3 Multivariate analysis for patients with NSTE-ACS

Multivariate logistic regression analysis identified NT-proBNP, D-dimer, and hs-CRP as independent predictors for the occurrence of MACE before revascularisation (Tables 3, 4). Specifically, NT-proBNP was independently associated with MACE (OR = 1.000, 95% CI: 1.000–1.000, *P* = 0.035), D-dimer demonstrated a significant predictive effect (OR = 1.916, 95% CI: 1.036–3.544, *P* = 0.038), and hs-CRP also remained an independent predictor (OR = 1.086, 95% CI: 1.023–1.152, *P* = 0.007).

**Table 3:** Screening out independent risk factors of MACE by multivariate analysis.

Factors	*Β*	OR	95% CI	*P*
Myo (ug/mL)	−0.002	0.998	0.933–1.004	0.501
CK-MB (IU/mL)	0.036	1.037	0.999–1.076	0.056
cTnI (ng/mL)	−0.387	0.679	0.364–1.265	0.222
NT-proBNP (pg/mL)	0.000	1.000	1.000–1.000	**0** **.** **034**
D-dimer (ug/mL)	0.867	2.380	1.138–4.979	**0** **.** **021**
HbAlc (%)	0.190	1.209	0.824–1.775	0.332
hs-CRP (mg/L)	0.126	1.134	1.040–1.236	**0** **.** **004**
Leu (×10^9^/L)	0.451	1.570	0.802–3.075	0.188
Neu (×10^9^/L)	−0.863	0.422	0.174–1.026	0.057
Scr (μmoL/L)	0.003	1.003	0.983–1.024	0.787

Myo, myohemoglobin; CK-MB, creatinine kinase MB; cTnI, cardiac Troponin I; NT-proBNP, N-terminal pro-B type natriuretic peptide; HbAlc, glycosylated hemoglobin; hs-CRP, hypersensitive C reactive protein; Leu, leukocyte; Neu, neutrophile granulocyte; Scr, serum creatinine.

Bold values indicates statistical significance (*p* < 0.05).

**Table 4:** Logistic multivariate analysis for establishing regression model.

Independent risk factors	*Β*	OR	95% CI	*P*
NT-proBNP (pg/mL)	0.000	1.000	1.000–1.000	**0** **.** **035**
D-dimer (ug/mL)	0.650	1.916	1.036–3.544	**0** **.** **038**
hs-CRP (mg/L)	0.082	1.086	1.023–1.152	**0** **.** **007**

NT-proBNP, N-terminal pro-B type natriuretic peptide; hs-CRP, hypersensitive C reactive protein.

Bold values indicates statistical significance (*p* < 0.05).

These detailed odds ratios, confidence intervals, and *P*-values have been added to ensure statistical transparency and facilitate interpretation (Table 4).

### 3.4 Comparison among different models for MACE prediction

**Figure 1:**
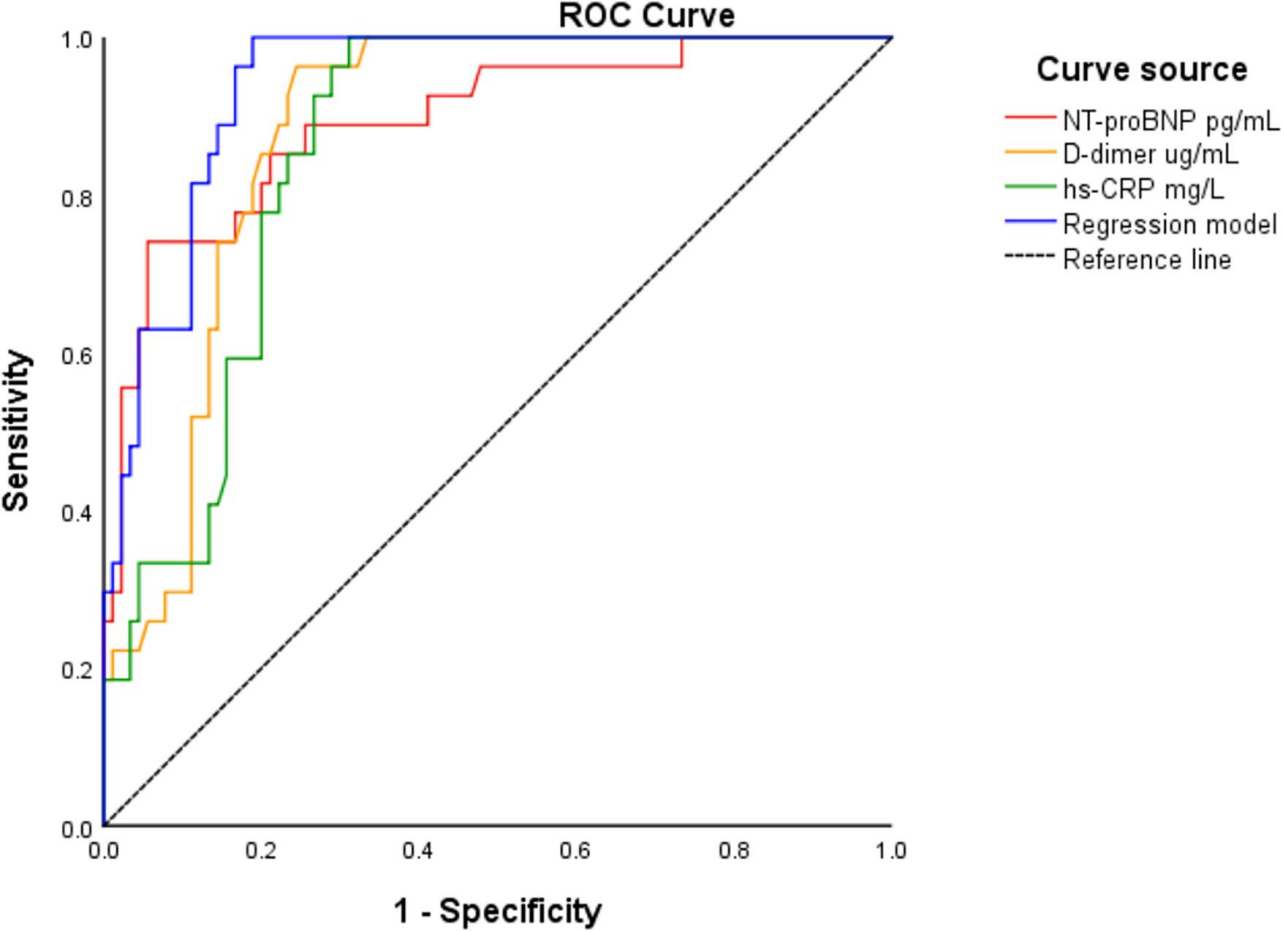
ROC analysis for NT-proBNP, D-dimer, hs-CRP and the regression model to predict MACE in patients with NSTE-ACS.

**Figure 2:**
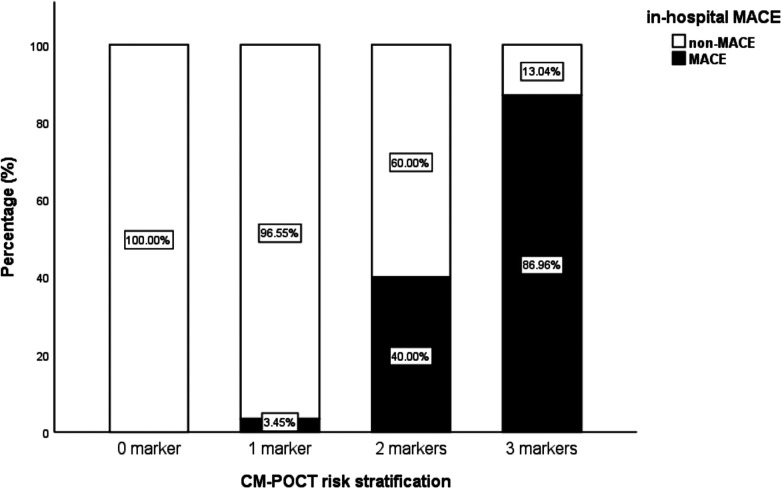
Incidences of MACE when the total number of the positive markers reached to 0–3.

To evaluate the predictive performance of the three independent biomarkers and the regression model, ROC curve analysis was used. The optimal cutoff values were as follows: NT-proBNP 2,410 pg/mL (sensitivity 74.1%, specificity 94.4%), D-dimer 0.54 μg/mL (sensitivity 96.3%, specificity 75.6%), and hs-CRP 5.58 µg/mL (sensitivity 100%, specificity 68.9%). The corresponding AUC values were 0.894 (95% CI: 0.821–0.966) for NT-proBNP, 0.881 (95% CI: 0.821–0.941) for D-dimer, and 0.860 (95% CI: 0.795–0.925) for hs-CRP—all statistically significant (*P* < 0.001). The regression model incorporating the three biomarkers demonstrated an AUC of 0.939 (95% CI: 0.899–0.979) (Figure 1). When the number of biomarkers exceeding the cutoff values increased, the incidence of MACE rose significantly (*P* < 0.001) (Figure 2).

**Figure 3:**
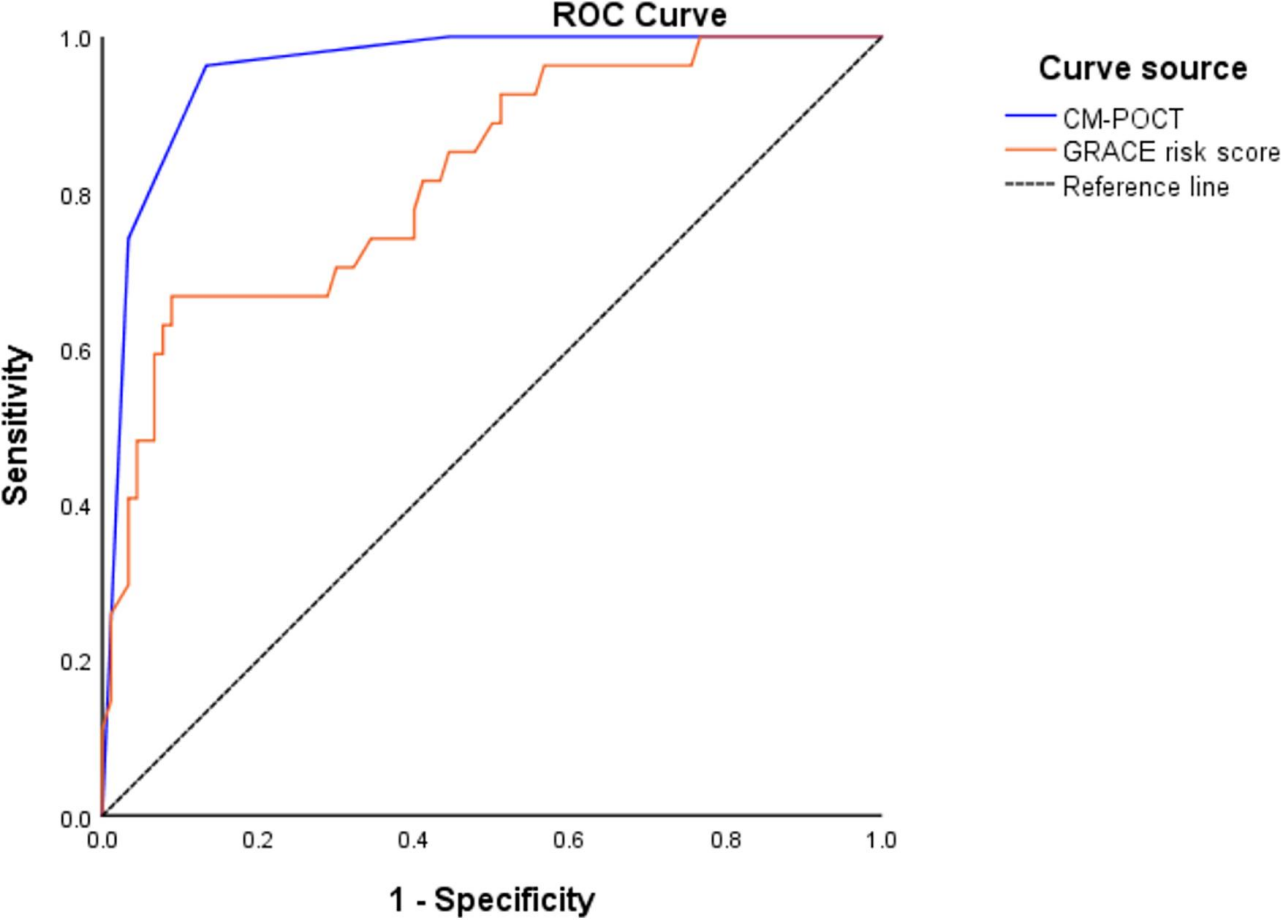
ROC curves of CM-POCT and GRACE risk score to predict MACE.

Further comparison showed that the CM-POCT marker panel achieved the highest discriminatory value with an AUC of 0.958 (95% CI: 0.923–0.993), significantly outperforming the GRACE risk score (AUC: 0.822, 95% CI: 0.730–0.913; *P* < 0.05). Although there was no significant difference between the GRACE risk score and each single biomarker (NT-proBNP, D-dimer, and hs-CRP), both the regression model and the CM-POCT panel showed superior predictive efficiency compared with the GRACE score (*P* < 0.05) (Figure 3, Table 5).

**Table 5:** Comparing with GRACE risk score to predict MACE.

Predictors	AUC	95% CI	*P*′	Cutoff-value of AUC	*D*-value of AUC	*P*
NT-proBNP	0.894	0.821–0.966	0.000	2,410 pg/mL	0.072	0.114
D-dimer	0.881	0.821–0.941	0.000	0.54 µg/mL	0.059	0.145
hs-CRP	0.860	0.795–0.925	0.000	5.58 µg/mL	0.038	0.254
Regression model	0.939	0.899–0.979	0.000	—	0.117	**0** **.** **011**
CM-POCT	0.958	0.923–0.993	0.000	—	0.136	**0** **.** **003**
GRACE risk score	0.822	0.730–0.913	0.000	—	0.000	—

*D*-value of AUC, the difference value with the AUC of GRACE risk score; NT-proBNP, N-terminal pro-B type natriuretic peptide; hs-CRP, hypersensitive C reactive protein; POCT, point-of-care testing; CM-POCT, combinative markers of POCT; GRACE, global registry of acute coronary events; *P*′, the difference compared with the baseline AUC = 0.5; *P*, the difference compared with the GRACE risk score (AUC = 0.822).

Bold values indicates statistical significance (*p* < 0.05).

## 4 Discussion

This study focused on patients aged ≥60 years with NSTE-ACS to explore the early predictive value of CM-POCT for MACE prior to revascularisation. We found that elevated NT-proBNP, D-dimer, and hs-CRP levels measured within 6 h of symptom onset during POCT were independent risk markers for MACE. Compared with the GRACE risk score, the CM-POCT demonstrated higher predictive efficacy, suggesting its potential as an early risk stratification tool for patients with NSTE-ACS in the ED.

In the MACE group, clinical and laboratory parameters—many of which are components of the GRACE risk score—such as age, heart rate, serum creatinine, Killip class, and cTnI, were significantly elevated compared with the non-MACE group, which is consistent with previous findings (7–9). Point-of-care testing allows for biomarker detection within 15 min (10, 11), making it a faster alternative to traditional risk scoring tools such as GRACE, which may require more comprehensive clinical data (17).

Our findings align with prior studies that identified NT-proBNP as an independent predictor of adverse outcomes in NSTE-ACS (10, 13, 18). N-terminal pro-B-type natriuretic peptide reflects myocardial wall stress and subclinical ventricular dysfunction caused by ischaemia-related pressure or volume overload; cardiomyocytes respond by upregulating BNP gene expression and releasing pro-hormone fragments into the circulation, indicating haemodynamic strain and impending heart failure (19, 20).

D-dimer indicates activation of the coagulation and fibrinolytic systems. Elevated D-dimer levels reflect ongoing thrombosis and fibrin degradation associated with plaque rupture or distal embolisation, thereby predisposing patients to recurrent ischaemic injury and haemodynamic compromise (21–25).

High-sensitivity C-reactive protein serves as a sensitive biomarker of systemic and vascular inflammation. Persistent inflammatory activation promotes endothelial dysfunction and macrophage infiltration within atherosclerotic plaques, and CRP deposition accelerates matrix breakdown and plaque instability, which can ultimately trigger acute coronary events (26, 27).

Together, these three biomarkers capture distinct but interrelated biological axes—ventricular strain (NT-proBNP), thrombosis (D-dimer), and inflammation (hs-CRP)—that converge to promote myocardial ischaemia and adverse outcomes in NSTE-ACS. This mechanistic integration underscores the rationale for using a multimarker CM-POCT strategy to identify high-risk patients before overt myocardial necrosis becomes evident.

These pathophysiological changes often precede detectable myocardial necrosis, making early biomarker-based risk assessment particularly valuable (18–20, 22–27). Compared with previous studies focusing on peak biomarker concentrations post-hospitalisation (15, 16, 28), our study highlights the clinical relevance of immediate ED-based testing. Especially during the diagnostic “grey zone” where repeated troponin testing or full risk scoring is still pending (6–9, 29, 30), CM-POCT offers clinicians a rapid and practical tool to identify high-risk elderly patients.

Furthermore, we demonstrated that each individual marker had reasonable predictive power for in-hospital MACE, and their combination significantly enhanced the overall predictive performance. The incidence of MACE increased in proportion to the number of markers exceeding cutoff thresholds, indicating that a simple count of positive results may serve as an intuitive and clinically feasible risk stratification method for patients aged ≥60 years with NSTE-ACS. This simplified counting approach effectively functions as a preliminary clinical algorithm within the CM-POCT framework. Future studies should further refine this concept into a validated scoring system by integrating biomarker data with established clinical variables.

Although this study specifically analysed events prior to revascularisation, it is important to acknowledge that adverse cardiovascular outcomes may also occur after PCI. Recent evidence has shown that periprocedural myocardial infarction is associated with significantly worse short- and long-term outcomes in patients with NSTEMI (31). Future studies should therefore evaluate whether point-of-care biomarkers also have prognostic value for periprocedural complications, which would further extend the clinical applicability and translational potential of the CM-POCT model. Moreover, since the study focused on patients before revascularisation, the CM-POCT model may serve as a useful tool for early and dynamic risk assessment during hospitalisation, potentially assisting clinicians in optimising the timing of elective PCI.

### 4.1 Limitations

Several limitations should be acknowledged. First, this was a retrospective, single-centre study with a relatively small sample size, particularly in the MACE group, which inherently limits the generalisability of the findings. Second, due to the retrospective design, we did not assess the dynamic changes in biomarkers or conduct long-term follow-up to evaluate their predictive value beyond hospitalisation. The analysis was restricted to in-hospital MACE prior to revascularisation, and no post-discharge or periprocedural events were recorded, which limits the evaluation of long-term prognostic utility. Third, the study exclusively enrolled elderly patients, and the results may not be generalisable to younger populations with NSTE-ACS. Finally, although the CM-POCT model demonstrated excellent discrimination (AUC = 0.958), this result should be interpreted with caution because of the small, single-centre cohort, which may lead to model overfitting. Before clinical translation, the model should undergo prospective multicentre validation and refinement to ensure stability and applicability across diverse emergency settings. Future multicentre studies with larger sample sizes and external validation are warranted to confirm the robustness and generalisability of the model.

## 5 Conclusion

In summary, our study demonstrated that the combined use of NT-proBNP, D-dimer, and hs-CRP measured early via CM-POCT provides complementary information for MACE risk stratification prior to revascularisation in patients aged ≥60 years with NSTE-ACS. This CM-POCT approach may facilitate more rapid and efficient decision-making in emergency settings compared with the GRACE risk score. Notably, the presence of multiple elevated markers was associated with a progressively higher risk of in-hospital MACE, supporting the utility of a simple marker count in clinical practice.

## Data Availability

The original contributions presented in the study are included in the article/Supplementary Material, further inquiries can be directed to the corresponding authors.

## Supplementary material

Supplementary Figure S1 The research flowchart.
