# Supplementary figures and images for: Combined point-of-care biomarkers for risk stratification in patients with non-ST-elevation acute coronary syndrome in the emergency department
Source: Front Cardiovasc Med. 2026 Feb 5;12:1711275. doi: 10.3389/fcvm.2025.1711275 (PMC12916683; doi:10.3389/fcvm.2025.1711275)

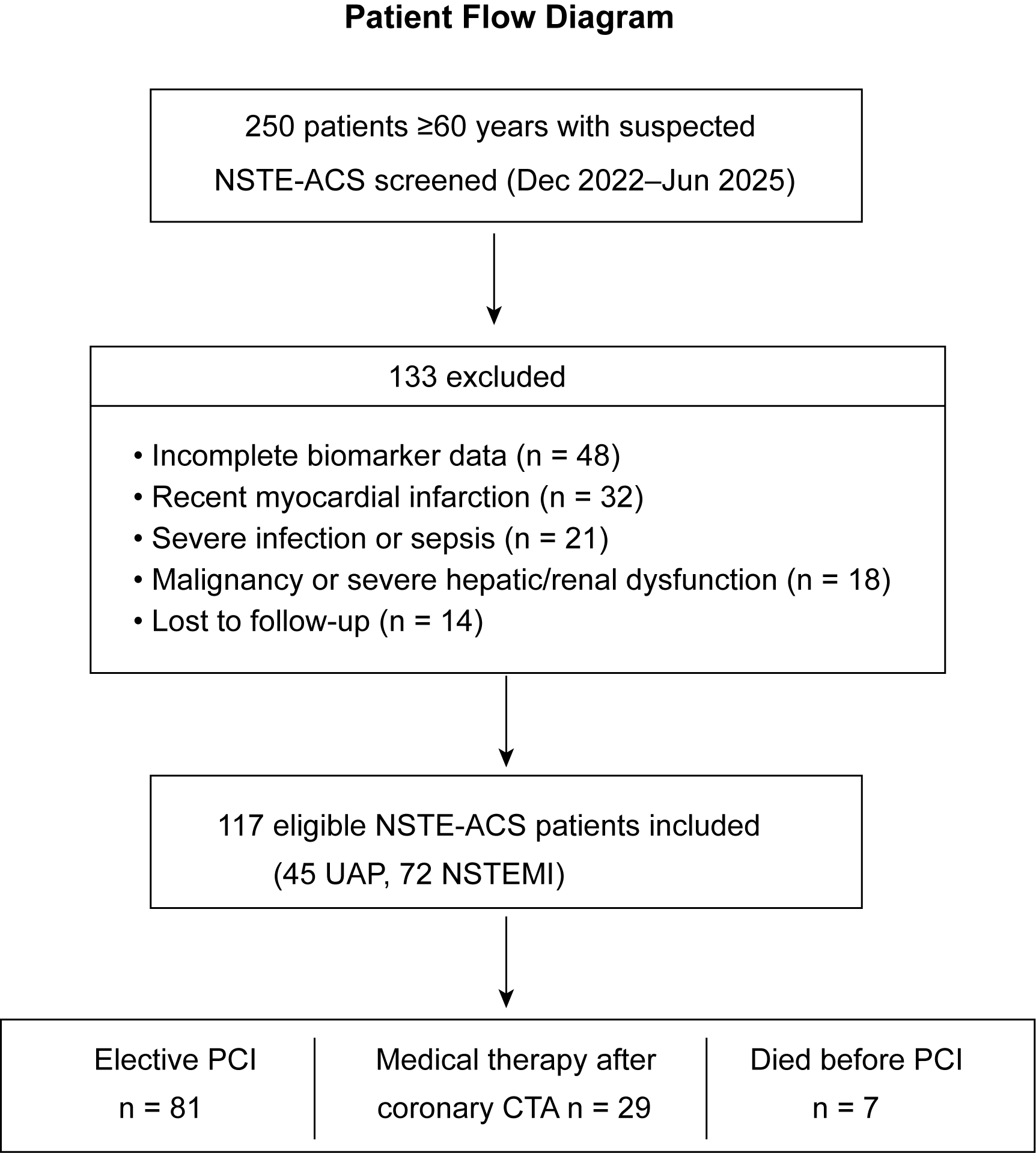

Supplement: Supplementary Figure S1 — The research flowchart. [file Image1.tif]
